# Inverse probability weighted estimation of dynamic treatment regimen means in sequential multiple assignment randomised trials with missing data: a simulation study

**DOI:** 10.1186/s13063-026-09493-x

**Published:** 2026-01-30

**Authors:** Jessica Xu, Robert K. Mahar, Katherine J. Lee, Anurika P. De Silva, Julie A. Simpson

**Affiliations:** 1 Centre for Epidemiology and Biostatistics, Melbourne School of Population and Global Health, University of Melbourne, Parkville, VIC Australia; 2 Clinical Epidemiology and Biostatistics Unit, Murdoch Children’s Research Institute, Parkville, VIC Australia; 3 Methods and Implementation Support for Clinical and Health (MISCH) Research Hub, Faculty of Medicine, Dentistry and Health Sciences, The University of Melbourne, Melbourne, VIC Australia; 4 Department of Paediatrics, University of Melbourne, Parkville, VIC Australia; 5 Nuffield Department of Medicine, University of Oxford, Oxford, UK

**Keywords:** Sequential multiple assignment randomised trials, Dynamic treatment regimens, Missing data, Inverse probability weighting, Multiple imputation

## Abstract

**Background:**

Dynamic treatment regimens (DTRs) guide personalised sequential treatment decisions for patients with a range of clinical or behavioural diseases. Sequential multiple assignment randomised trials (SMARTs) are designed to evaluate and optimise DTRs by randomising participants at multiple stages based on intermediate outcomes. To identify optimal DTRs in SMARTs, the mean outcome of each DTR is often estimated via inverse probability weighting (IPW), a statistical method that uses the inverse probability of treatment to address potential bias in the design. Like other randomised controlled trials, SMARTs are subject to missing data. Handling missing data in SMARTs is complicated by the sequential randomisation and dependence on intermediate outcomes. We evaluated the performance of complete case analysis (CCA) and multiple imputation (MI) for handling missing data when estimating the DTR mean outcomes using IPW in a two-stage SMART.

**Methods:**

We simulated 1000 datasets of 400 participants, based on a prototypical SMART design with two stages where only non-responders are re-randomised at stage 2. The estimands of interest were the four DTR means of a continuous outcome and were estimated using IPW. We defined four plausible missing data scenarios using missing data directed acyclic graphs (m-DAGs) and then assessed how each missing data method (CCA and MI) performed under different proportions of missingness (20%, 40%) and strengths of associations with missingness in stage 1 intermediate outcome, stage 2 treatment, and the final outcome.

**Results:**

Minimal bias was observed with MI when estimating the mean outcomes of the DTRs in most scenarios, except for when stage 1 intermediate outcome was missing dependent on baseline variables and stage 1 treatment. When data were missing dependent on other variables (for example, stage 2 treatment missing dependent on stage 1 intermediate outcome), CCA generally showed greater bias than MI when estimating the mean outcomes of the DTRs. Empirical standard errors were comparable across both missing data methods, with MI generally producing slightly lower values.

**Conclusion:**

We found that for a prototypical SMART design, MI generally showed close to zero bias and slightly lower standard errors compared to CCA when IPW was used to estimate the mean outcomes of DTRs in the settings explored.

**Supplementary Information:**

The online version contains supplementary material available at 10.1186/s13063-026-09493-x.

## Introduction

Dynamic treatment regimens (DTRs) are a set of sequential treatment rules used to guide multi-stage clinical decisions by specifying how treatment choices adapt over time based on patient's disease progression and on ongoing patient response to a range of clinical or behavioural diseases [1–4]. Sequential multiple assignment randomised trials (SMARTs) are multi-stage studies used to evaluate and optimise DTRs. SMARTs randomise patients repeatedly over time to different treatments that depend on intermediate outcomes. A SMART embeds a number of DTRs [5, 6], the aim typically being to identify the DTR with the optimal average outcome.

Estimating outcomes for each embedded DTR and identifying the optimal DTR in a SMART requires complicated statistical approaches due to the interplay between patient histories, sequential treatment allocation probabilities, and outcomes. Statistical methods commonly used include g-computation [7], Q-learning [8], and weighted regression methods such as inverse probability weighting (IPW) [9, 10]. IPW is a method that uses weights to account for the varying probabilities of treatment assignments at each stage to estimate the population mean outcome for each DTR. The weights are calculated as the inverse of the probability of receiving each participant’s observed sequence of treatment, based on the known randomisation probabilities at each stage. In a scoping review, IPW was found to be the most frequently used approach for determining optimal DTRs in observational data [2]. Studies have shown that IPW is also commonly used to estimate the mean outcome of the embedded DTRs in SMARTs, which then can be used in different DTR comparisons [11, 12].

Like all randomised controlled trials, missing data is a problem in SMARTs that complicates the data analysis. Because SMARTs consist of multiple stages of randomisation based on intermediate responses to treatment at each stage, SMARTs can have more complicated patterns of missingness compared to standard randomised controlled trials. For example, in the Clinical Antipsychotic Trials of Intervention Effectiveness (CATIE) SMART, out of the 1460 participants who were randomised to a treatment at stage 1, 705 completed the study and 744 (52%) dropped out before the end of the study [13]. In the Staged Treatment in Early Psychosis (STEP) 3-stage SMART, 61 out of 342 (17.8%) participants dropped out before the end of stage 1, another 134 out of 281 (47.7%) participants who were randomised to a treatment at stage 2 (dependent on their remission status) dropped out at the end of stage 2 and did not go on to stage 3. An extra 53 out of 138 (38.4%) participants without remission who were randomised in stage 3 dropped out before the end of stage 3 [14]. In another SMART that included minimally verbal school-aged children with autism [15], out of the 61 participants who were randomised to a treatment at stage 1, 6 (10%) dropped out before progressing to stage 2, and another 9 dropped out before the end of stage 2, leading to a total dropout rate of 25%. Understanding the underlying reasons for missing data and using appropriate statistical methods to handle missing data are important to ensure valid analyses [16]. Common methods used to handle missing data include complete case analysis (CCA) and multiple imputation (MI). In a CCA, participants with missing data for any of the analysis variables are excluded [17]. MI is a two-stage process that imputes missing values multiple times based on observed data, analyses each imputed dataset, and combines the results while accounting for uncertainties associated with the missing values [18].

Although many studies have explored how CCA and MI perform when handling missing data in typical single-stage randomised trials [19, 20], there is limited literature on their performance in SMARTs [21, 22]. Shortreed et al. [21] explored how well CCA and MI performed in a SMART setting when weighted regression was used to estimate the mean response for each DTR using data from a case study. Their work provided an important demonstration of how MI can be applied in practice. However, no simulation study was conducted, and it remains unclear how CCA and MI perform when missingness arises from different missing data scenarios in a SMART setting. Following this, we conducted a simulation study to evaluate the performance of CCA and MI in a simple two-stage, two-treatment SMART on the estimation of the quality of stage-wise treatment decisions in SMARTs using Q-learning [22]. We found that MI performed poorly compared to CCA, which we assume is due to the backward induction nature of Q-learning leading to incompatibilities between imputation and analysis models.

In this study, we evaluated the performance of CCA and MI for handling missing data when estimating the mean outcomes of the embedded DTRs using IPW in a two-stage, two-treatment SMART where stage 2 treatment depends on the intermediate outcome. We begin by formalising four missing data scenarios that might be observed in a SMART using missing data directed acyclic graphs (m-DAGs) [16, 23]. Next, we review IPW as a method for estimating the mean outcome in DTRs and describe in detail the simulation study [24] we conducted to examine the performance of CCA and MI under a range of missingness proportions and missing data mechanisms. We then present the results of the simulation study and end with conclusions and general recommendations.

## Methods

### Missingness in SMART designs

**Fig. 1:**
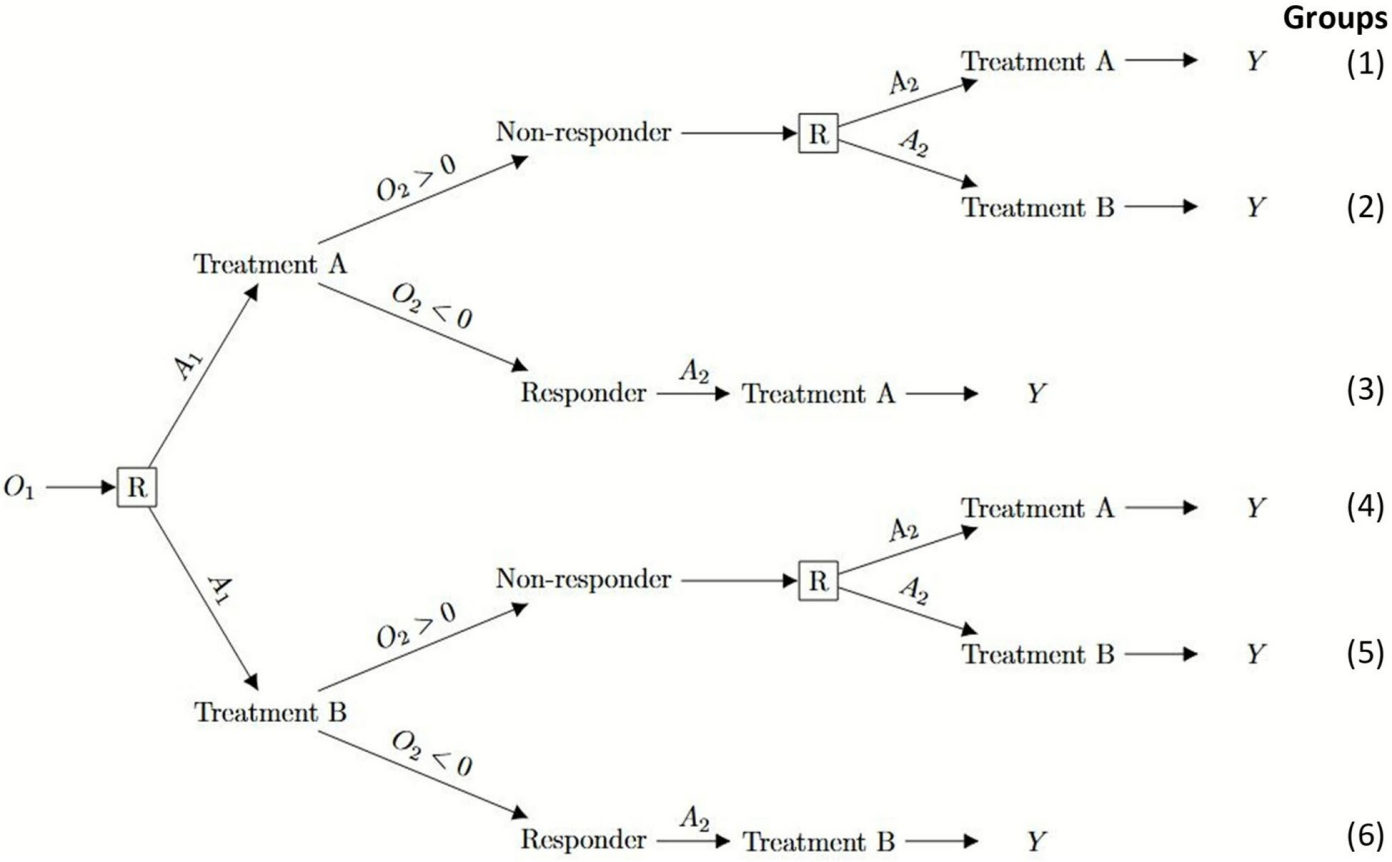
Example of a two-stage SMART design where only non-responders are re-randomised at stage 2. ${O}_{1}$ represents a baseline variable; ${A}_{1}$ and ${A}_{2}$ represent treatment at stage 1 and stage 2, respectively; ${O}_{2}$ represents the intermediate outcome, where a participant is considered a non-responder if ${O}_{2}>0$; and $Y$ represents the outcome after stage 2 treatment (also the final outcome). R is randomisation at each stage

A common two-stage SMART design [25] is presented in Fig. 1. In this design, eligible participants are randomised between two treatments at stage 1. Responders to stage 1 treatment stay on the same treatment and only non-responders are re-randomised at stage 2. We denote a continuous baseline variable by ${O}_{1}$; treatments (binary) at stage 1 and stage 2 by ${A}_{1}$ and ${A}_{2}$, respectively; a continuous intermediate outcome by ${O}_{2}$, where participants are considered as non-responders if ${O}_{2}>0$; and the continuous outcome after stage 2 treatment by $Y$, which we refer to as the final outcome. We assume that participants with missing ${O}_{2}$ dropped out of the study and therefore have missing treatment at stage 2 (${A}_{2}$) and outcome ($Y$).

In the SMART design shown in Fig. 1, there are 4 embedded DTRs: (1) treatment A at stage 1, followed by treatment A at stage 2 for both responders and non-responders; (2) treatment A at stage 1, followed by treatment A for responders and treatment B for non-responders at stage 2; (3) treatment B at stage 1, followed by treatment B at stage 2 for both responders and non-responders; (4) treatment B at stage 1, followed by treatment B for responders and treatment A for non-responders at stage 2. In some literature [26], DTRs (1) and (3) are referred to as static treatment regimens, as participants remain on the same treatment for both stages.

**Fig. 2:**
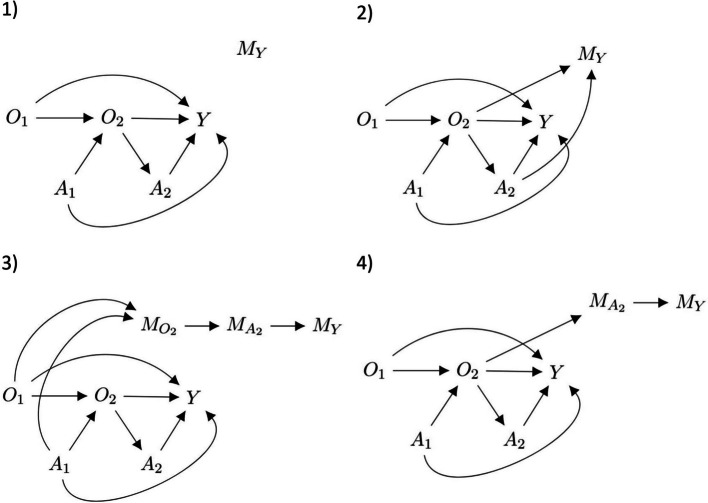
m-DAGs depicting a two-stage SMART with missing data according to four missingness scenarios. The nodes ${M}_{O2}$, ${M}_{A2}$ and ${M}_{Y}$ represent missingness in ${O}_{2}$, ${A}_{2}$ and $Y$, respectively. When ${O}_{2}$ is missing, both ${A}_{2}$ and $Y$ are also missing

When there is missing data in SMARTs, m-DAGs can be used to describe the missingness. Figure 2 shows the four missingness scenarios that we might observe within the two-stage, two-treatment SMART design in Fig. 1. The nodes ${M}_{O2}$, ${M}_{A2},$ and ${M}_{Y}$ in the m-DAG [16, 23] represent missing data in ${O}_{2}$, ${A}_{2},$ and $Y$, respectively. We assume that ${O}_{1}$ and ${A}_{1}$ are complete.

- Missing data scenario 1: Data records for the final outcome could be lost post data collection at the end of stage 2, which would lead to the final outcome ($Y$) to be missing not dependent on any variables.
- Missing data scenario 2: The final outcome ($Y$) could be missing dependent on the intermediate outcome at stage 1 (${O}_{2}$) and the treatment given in stage 2 (${A}_{2}$). For example, participants who were non-responders (${O}_{2}>0$) to stage 1 treatment and re-randomised to a less effective treatment arm at stage 2 could be more likely to drop out before the final outcome data are collected.
- Missing data scenario 3: Participants could drop out after stage 1 leading to missing intermediate outcome at stage 1 (${O}_{2}$), missing treatment assignment in stage 2 (${A}_{2}$), and missing outcome data in stage 2 ($Y$). This could occur for many reasons including concerns about potential side effects, dissatisfaction with the treatments or baseline characteristics (i.e. dropping out due to age or time constraints). Reasons for drop out could be related to stage 1 treatment (${A}_{1}$) and baseline variables (${O}_{1}$).
- Missing data scenario 4: Participants who were non-responders (${O}_{2}>0$) to stage 1 treatment may be more likely to drop out prior to stage 2 and therefore have missing treatment assignment (${A}_{2}$) and outcome at stage 2 ($Y$). In this scenario, ${A}_{2}$ could be missing dependent on ${O}_{2}$.

We only considered scenarios where participants drop out after stage 1 (missing data scenario 3) or prior to stage 2 (missing data scenario 4). We did not consider scenarios where participants have missing ${O}_{2}$ and remain in the study as we expect this to be very unlikely in practice.

### Inverse probability weighting

Inverse probability weighting (IPW) is a commonly used method for estimating the mean outcome in DTRs, both in observational studies [2] and in SMARTs [11, 12]. The IPW approach weights each individual’s outcome by the (inverse) probability that the individual received their sequence of treatments. To demonstrate the principle in general, assume we run our example SMART (Fig. 1) with equal randomisation probabilities at each stage (conditional on history). If we want to evaluate the average outcome of participants who have data consistent with a specific DTR, bias arises due to the study design because responders are not re-randomised, while non-responders are randomised to two subsequent treatments (i.e. randomised between 2 different DTRs). As a result, the dataset will include twice as many participants who are responders compared to non-responders for each DTR (because half will be randomised to a different DTR). Consequently, the calculated average outcome of a DTR will be overrepresented by the outcomes of responders. To ensure that non-responders are equally represented within each DTR, we assign non-responders twice the weight of responders.

For a two-stage, two-treatment SMART, we define the stages by $j$, where $j=1, 2$. We define ${A}_{j}$ as treatment assignment at stage $j$; ${\overline{A} }_{j}$ as the treatment assignment history up to stage $j$; $R$ as a response indicator to stage 1 treatment; and $Y$ as the observed continuous outcome at the end of the final stage. Let ${\theta }_{k}=E\left[Y\left({d}_{k}\right)\right]$ be the population mean of the outcome $Y$ when all participants follow the $k$th DTR (defined as ${d}_{k}$) and $N$ be the total number of DTRs embedded within the design, where $k=1,\dots , N$. To estimate ${\theta }_{k}$, we define inverse probability weights for each combination of treatment history and responder status as follows:

1 $$w\left({\overline{A} }_{2},R\right)=\frac{{I}_{k}({A}_{1}){I}_{k}({A}_{2})}{P\left({A}_{1}={a}_{1}\right)P({A}_{2}={a}_{2}|{A}_{1}={a}_{1}, R=r)}$$

For Eq. 1, the indicator functions ${I}_{k}\left({A}_{1}\right)$ and ${I}_{k}\left({A}_{2}\right)$ equal 1 if the participant’s treatment assignments at stages 1 and 2, respectively, are consistent with the $k$th DTR, and 0 otherwise. The indicator functions ensure that the correct weights are applied only to participants whose observed treatment paths match the DTR being evaluated. For each participant, we calculate the inverse of the probability receiving their actual sequence of treatments, based on the known randomisation probabilities.

For the analysis, we specify a marginal structural model (MSM) for the population mean of the DTR outcomes, to match the specified SMART design. Using the calculated weights from Eq. 1, we perform a weighted regression to estimate the parameters of the MSM [9, 27].

### Simulation study

We performed a simulation study to evaluate the performance of CCA and MI for handling missing data in a two-stage SMART when estimating the mean outcome of each of the embedded DTRs using IPW. We considered the same simulation settings used by Ertefaie et al. [9], whose simulation study focused on identifying a list of DTRs that contain the best DTR or several optimal DTRs. Of note, their simulation study did not explore missing data and thereby evaluate or consider methods for handling missing data.

#### Data generation

We simulated 1000 datasets each with a sample size of 400 participants, using the data-generating models described below:

1. Baseline variable ${O}_{1}$ was generated from a standard normal distribution $N(\mathrm{0,1})$.
2. Participants were randomly assigned a treatment (binary) at stage 1 with the probability of 0.5:
  2 $$P({A}_{1} = 1) = P({A}_{1} = -1) = 0.5$$
3. The intermediate outcome of stage 1 collected prior to stage 2 was generated conditional on the baseline variable and stage 1 treatment: ${O}_{2}\sim N\left(0.5{O}_{1} + 0.5 {\it\mathrm{I}}\left({A}_{1}=-1\right),1\right).$
4. A binary stage 1 responder status ($R$) was generated to indicate whether a participant was a responder to stage 1 treatment. If ${O}_{2}<0$, the participant was considered a responder ($R=1$).
5. Non-responders to stage 1 treatment were randomly assigned to a treatment at stage 2 with the probability of 0.5 (as responders only have one treatment option, we do not include ${A}_{2}^{R}\tt ):$
  3 $$P({A}_{2}^{NR} = 1) = P({A}_{2}^{NR} = -1) = 0.5$$
6. Stage 2 outcome (continuous) was generated as $Y =E\left(Y \right| {O}_{1}, {A}_{1}, {O}_{2}, {A}_{2}^{NR}).$
  4 $$Y=1+{O}_{1}+{O}_{2}+{A}_{1}\left(\delta +{O}_{1}\right)+S(\delta /2){A}_{2}^{NR}+\epsilon , \epsilon \sim N\left(\mathrm{0,1}\right)$$

where $S=I({O}_{2}>0)$ indicates a non-responder ($R=0$) who is re-randomised and $\delta$ = 0.1 was the effect of treatment B compared to treatment A at both stages and at stage 2 this is for non-responders only.

#### Missingness

We considered the 4 missing data scenarios described above (Fig. 2). All variables not mentioned in a scenario were considered fully observed. Data were set to missing as follows where ${M}_{Y}$, ${M}_{O2},$ and ${M}_{A2}$ represent indicators for missing data in the outcome ($Y$), intermediate outcome after stage 1 (${O}_{2}$) and treatment at stage 2 (${A}_{2}$), respectively:

1. Missing data scenario 1: stage 2 outcome ($Y$) was set to missing not dependent on any variables.
2. Missing data scenario 2: stage 2 outcome data ($Y$) were set to missing dependent on the stage 1 intermediate outcome ${O}_{2}$ and stage 2 treatment for non-responders ${A}_{2}^{NR}$ using the following logistic regression model:
  5 $${\mathrm{logit}}[P({M}_{Y} = 1)] = {\alpha }_{0} + {\alpha }_{1}{O}_{2}+{\alpha }_{2}[{A}_{2}^{NR} = 1]$$
3. Missing data scenario 3: stage 1 intermediate outcome (${O}_{2}$) was set to missing using a logistic regression model where the probability of missingness was dependent on the baseline variable (${O}_{1}$) and treatment at stage 1 (${A}_{1}$):
  6 $${\mathrm{logit}}[P({M}_{02} = 1)] = {\alpha }_{0} + {\alpha }_{1}{O}_{1} + {\alpha }_{2}[{A}_{1} = 1]$$
  In this scenario, stage 2 treatment (${A}_{2}^{NR}$) and the outcome ($Y$) were set to missing whenever there were missing data in the stage 1 intermediate outcome (${O}_{2}$).
4. Missing data scenario 4: if a participant was a non-responder to stage 1 treatment (${O}_{2}> 0$) then they were set to be more likely to have missing data for stage 2 treatment and outcome. This was achieved using the following logistic regression model:
  7 $${\mathrm{logit}}[P({M}_{A2} = 1)] = {\alpha }_{0} + {\alpha }_{1}{O}_{2}$$

For all 4 missing data scenarios mentioned above, we explored both weak and strong associations between the predictors of missingness and missingness indicator and explored both 20% and 40% missingness. For a weak association an odds ratio (OR) of 1.6 was used and for a strong association an OR of 3 was used. The intercepts of the logistic regression models (${\alpha }_{0})$ (Eqs. 5 to 7) above were chosen by iteration to achieve the required percentage of missingness (20% or 40%).

#### Estimands of interest and target analysis

Our interest lies in estimating the DTR mean outcomes (${\theta }_{k}$) of a two-stage, two-treatment SMART using IPW.

We set the MSM to be:

8 $$m\left(\beta \right)={\beta }_{0}+{\beta }_{1}{A}_{1}+{\beta }_{2}{A}_{2}^{NR}$$

By design, subgroups of participants were consistent with more than one DTR. For example, in our SMART design (Table 1) group 3 was consistent with both DTR1 and DTR2. To account for this issue and prevent bias, we prepare the data by replicating participants who were consistent with more than one DTR before the estimation process to estimate the mean outcome in each of the DTRs simultaneously with standard software [12]. Responders to stage 1 treatment were not assigned a treatment at stage 2 ($${A}_{2})$$. We duplicated the responders’ rows and assigned each row a treatment at stage 2, one row with $${A}_{2}=-1$$ and the other with $${A}_{2}=1$$. Each row therefore corresponded to a participant in a single DTR.

**Table 1:** Stage 1 (${A}_{1}$) and stage 2 treatment (${A}_{2}$) sequence for the four dynamic treatment regimens (DTRs) in Fig. 1

**DTR k**	${{\boldsymbol{A}}}_{1}$	**Stage 1 responder status**	${{\boldsymbol{A}}}_{2}$	**Group**
DTR 1	1	Non-responder	1	(1)
	1	Responder		(3)
DTR 2	1	Non-responder	−1	(2)
	1	Responder		(3)
DTR 3	−1	Non-responder	1	(4)
	−1	Responder		(6)
DTR 4	−1	Non-responder	−1	(5)
	−1	Responder		(6)

Since only a proportion of participants are randomised at the second stage, we need to use weights in our estimation. [28] For our SMART design, only the non-responders were randomised twice, so their weights were $1/(0.5*0.5)=4$. Responders were only randomised once, hence their weights were $1/0.5=2$. After replicating and weighting our data, estimation was done by using the *geeglm* function in R that incorporates generalised estimating equations estimators on the replicated dataset, assuming an independent covariance structure.

The mean outcome ${\theta }_{k}$ for each DTR was then estimated using the following:

9 $${\mathrm{DTR}}1: {\theta }_{1}={\beta }_{0}+{\beta }_{1}\left(1\right)+{\beta }_{2}\left(1\right)={\beta }_{0}+{\beta }_{1}+{\beta }_{2}$$

10 $${\mathrm{DTR}}2: {\theta }_{2}={\beta }_{0}+{\beta }_{1}\left(1\right)+{\beta }_{2}\left(-1\right)={\beta }_{0}+{\beta }_{1}-{\beta }_{2}$$

11 $${\mathrm{DTR}}3: {\theta }_{3}={\beta }_{0}+{\beta }_{1}\left(-1\right)+{\beta }_{2}\left(1\right)={\beta }_{0}-{\beta }_{1}+{\beta }_{2}$$

12 $${\mathrm{DTR}}4: {\theta }_{4}={\beta }_{0}+{\beta }_{1}\left(-1\right)+{\beta }_{2}\left(-1\right)= {\beta }_{0}-{\beta }_{1}-{\beta }_{2}$$

The true ${\beta }^{*}$(${\beta }_{0}$, ${\beta }_{1},$ and ${\beta }_{2}$) parameter values in the MSM (Eq. 8) were estimated by averaging the ${\beta }^{*}$ estimates over 1000 simulated datasets each with 10,000 participants which was found to be approximately ${\beta }^{*}=\left(1.25, -0.15, 0.03\right)$ and the true $\theta$ values for the four DTRs (derived from multiplying the true $\beta$ parameters with the relevant linear combinations of ${A}_{1}$ and ${A}_{2}$) were ${\theta }^{*}=\left(1.127, 1.069, 1.429, 1.372\right).$

#### Methods to handle missing data

We compared the performance of CCA and MI for handling missing data in each of the scenarios described above. For CCA, only participants with complete data for all the variables were included in the analysis. As we needed to impute both continuous and binary variables, MI by chained equations [29] was used to impute the missing values. Linear regression models were used to impute the continuous variables and logistic regression models were used to impute the binary variables. The imputation model for $$Y$$ (final stage outcome) included the baseline variable ($${O}_{1})$$, treatment variables ($${A}_{1}, {A}_{2}$$) and stage 1 intermediate outcome ($${O}_{2}$$). The imputation model for $${O}_{2}$$ included the baseline variable ($${O}_{1}$$), treatment variables ($${A}_{1}, {A}_{2}$$), and outcome ($$Y$$). The imputation model for $${A}_{2}$$ included the baseline variable ($${O}_{1}$$), treatment variable at stage 1 ($${A}_{1}$$), stage 1 intermediate outcome ($${O}_{2}$$), and outcome ($$Y$$). The number of imputations was determined based on the proportion of missing data [30]. For the scenarios with 20% missing data, 20 imputed datasets were generated, and 40 imputed datasets were generated when approximately 40% of the data were missing. We ran 5 iterations for each imputed dataset.

#### Performance measures

Performance measures selected to compare the performance of CCA and MI were the absolute bias, the difference between the average estimate over 1000 simulated datasets and the true value of the $\theta$ (or $\beta$) parameters; empirical standard errors, the square root of the empirical variance; bootstrap model-based standard errors, calculated by obtaining the standard error of the 200 bootstrap samples for each simulated dataset and then averaging over the 1000 simulated datasets; and coverage, the proportion of 95% confidence intervals that include the true value of the $\theta$ (or $\beta$) parameters.

All data simulation and analyses were conducted using R version 4.4.0.

## Results

**Fig. 3:**
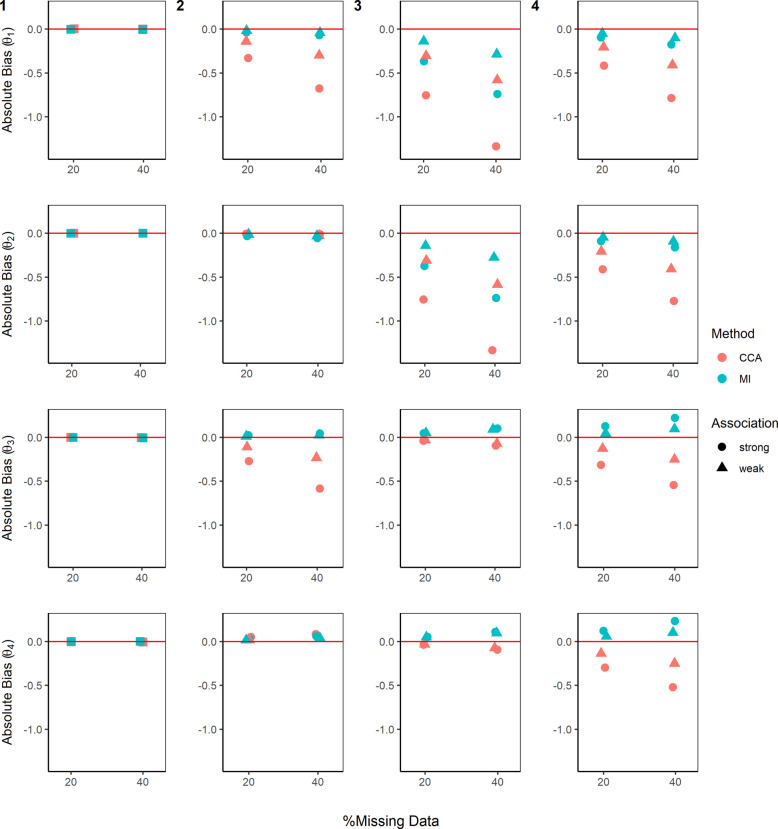
Absolute bias in estimating the mean outcome of the four dynamic treatment regimens ($\theta$). True values for mean outcome for DTR1–DTR4 are ${\theta }^{*}=\left(1.127, 1.069, 1.429, 1.372\right)$. Complete case analysis (CCA) and multiple imputation (MI) were used to handle missing data, where 20% or 40% had incomplete data under the four missing data scenarios (presented by columns) described in the Missingness in SMART designs section, see Fig. 2. For m-DAG 1, where only stage 2 outcome was missing and missingness was not dependent on any variables, a square symbol is used. For a weak association between the missing indicator and other variables (as described below) an OR of 1.6 was used, and for a strong association, an OR of 3 was used. The other variables used in missing data scenario: 2)${O}_{2}\to {M}_{Y}$ and ${A}_{2}\to {M}_{Y}$; 3) ${A}_{1}\to {M}_{O2}$ and ${O}_{1}\to {M}_{O2}$; and 4) ${O}_{2}\to {M}_{A2}$. Monte Carlo errors ranged from 0.0044 to 0.0100

**Fig. 4:**
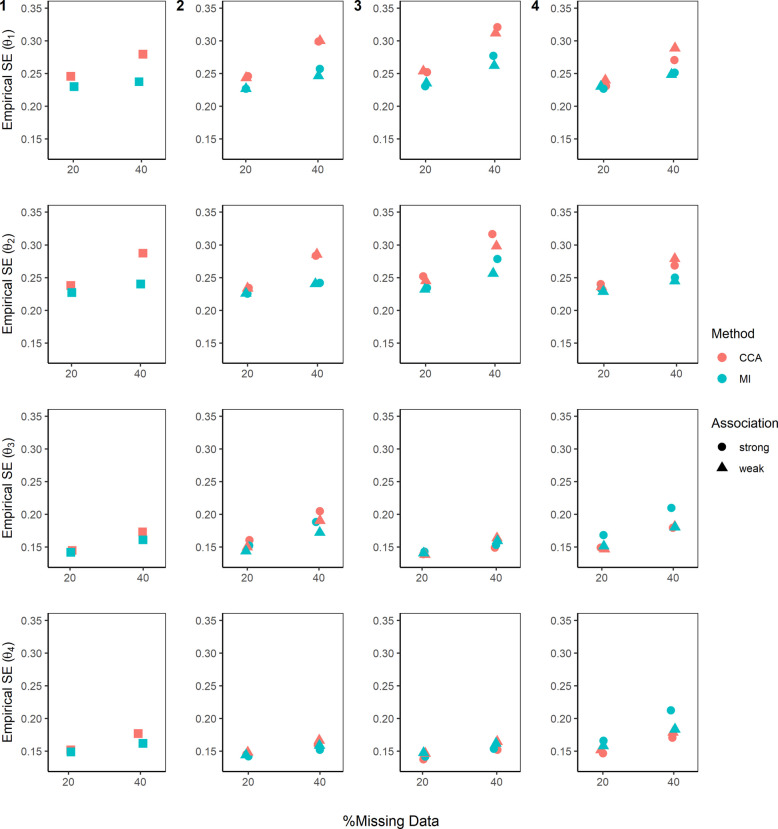
Empirical standard errors (SEs) for the mean outcome of the four dynamic treatment regimens ($\theta$). Complete case analysis (CCA) and multiple imputation (MI) were used to handle missing data, where 20% or 40% had incomplete data under the four missing data scenarios (presented by columns) described in the Missingness in SMART designs section, see Fig. 2. For m-DAG 1, where only stage 2 outcome was missing and the missingness was not dependent on any variables, a square symbol is used. For a weak association between the missing indicator and other variables (as described below) an OR of 1.6 was used, and for a strong association, an OR of 3 was used. The other variables used in missing data scenario: 2) ${O}_{2}\to {M}_{Y}$ and ${A}_{2}\to {M}_{Y}$; 3) ${A}_{1}\to {M}_{O2}$ and ${O}_{1}\to {M}_{O2}$; and 4) ${O}_{2}\to {M}_{A2}$. Monte Carlo errors ranged from 0.0031 to 0.0072

**Fig. 5:**
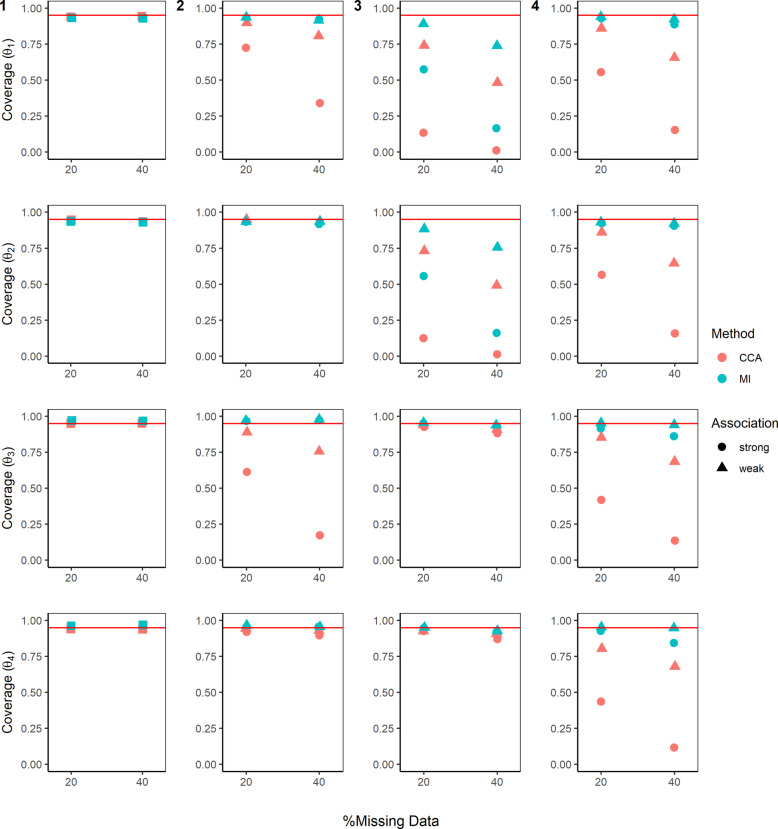
Coverage for the mean outcome of the four dynamic treatment regimens ($\theta$). Complete case analysis (CCA) and multiple imputation (MI) were used to handle missing data, where 20% or 40% had incomplete data under the four missing data scenarios (presented by columns) described in the Missingness in SMART designs section, see Fig. 2. For m-DAG 1, where only stage 2 outcome was missing and the missingness was not dependent on any variables, a square symbol is used. For a weak association between the missing indicator and other variables (as described below) an OR of 1.6 was used; and for a strong association an OR of 3 was used. The other variables used in missing data scenario: 2) ${O}_{2}\to {M}_{Y}$ and ${A}_{2}\to {M}_{Y}$; 3) ${A}_{1}\to {M}_{O2}$ and ${O}_{1}\to {M}_{O2}$; and 4) ${O}_{2}\to {M}_{A2}$. Monte Carlo errors ranged from 0.0030 to 0.0158

Figures 3, 4, and 5, Additional file 1: Figures S1–S3, and Additional file 2: Table S1 summarise the performance of CCA and MI for estimating the parameters of the MSM ($\beta$) and the DTR mean outcomes ($\theta$) across the different missing data scenarios described above.

### Absolute bias 

When estimating the mean outcome for each DTR (the $\theta$ parameters for missing data scenario 1 when stage 2 outcome was missing not dependent on any variables (m-DAG1, Fig. 2), we observed close to zero bias for both CCA and MI (Fig. 3).

For missing data scenario 2, when the outcome was missing dependent on the intermediate outcome at stage 1 and the treatment given in stage 2 (m-DAG2, Fig. 2), we observed close to zero bias for MI when estimating the mean outcomes for all four DTRs. However, for CCA we observed bias when estimating the mean outcome for DTR1 (${\theta }_{1}$) and DTR3 (${\theta }_{3}$) and no bias for DTR2 (${\theta }_{2}$) and DTR4 (${\theta }_{4}$). As shown in Figure S1, CCA estimation of ${\beta }_{1}$ had close to zero bias for m-DAG2 whereas bias was observed for both ${\beta }_{0}$ and ${\beta }_{2}$. Based on Eqs. 9–12, this bias cancelled out under DTR2 and DTR4 but accumulated under DTR1 and DTR3.

For missing data scenario 3, where the stage 1 intermediate outcome was missing dependent on baseline variables and stage 1 treatment (m-DAG3, Fig. 2), both CCA and MI showed bias when estimating the mean outcome for DTR1 (${\theta }_{1}$) and DTR2 (${\theta }_{2}$) but close to zero bias when estimating the mean outcome for DTR3 (${\theta }_{3}$) and DTR4 (${\theta }_{4}$). The lack of bias observed for DTR3 and DTR4 was likely due to similar reasons as explained above, the bias of ${\beta }_{0}$ and ${\beta }_{1}$ cancelling each other out from the linear combinations of ${A}_{1}$ and ${A}_{2}^{NR}$ (see Figure S1, Additional file 1 and Eqs. 9–12).

For missing data scenario 4, where stage 2 treatment was missing dependent on the intermediate outcome at stage 1 (m-DAG4, Fig. 2), we observed minimal bias for MI and greater bias for CCA when estimating the mean outcomes for all four DTRs.

When estimating both the $\beta$ and $\theta$ parameters, for all scenarios where we observed some bias, CCA showed greater bias than MI, and the bias increased with the proportion of missingness and the strength of the associations between the missing indicators and their predictors of missingness.

### Empirical standard errors and model-based standard errors

When estimating the mean outcome for each DTR (i.e., the $\theta$ parameters), for all scenarios, empirical SEs for $\theta$(and $\beta$) parameters were similar to their corresponding model-based SEs (Fig. 4; Figure S2, Additional file 1 and Table S1, Additional file 2).

We observed similar SEs when estimating the mean outcome for DTR1 (${\theta }_{1}$) and DTR2 (${\theta }_{2}$) which were higher than those observed in DTR3 (${\theta }_{3}$) and DTR4 (${\theta }_{4}$). This pattern can be explained by examining the variance components derived from linear combinations of ${A}_{1}$ and ${A}_{2}^{NR}$. All DTR variances involve the sum of the variances of ${\beta }_{0}$, ${\beta }_{1}$, and ${\beta }_{2}$ and their covariances. While the variances of ${\beta }_{0}$, ${\beta }_{1}$, and ${\beta }_{2}$ contribute similarly across all the DTRs, there is a difference in the term for the covariance between ${\beta }_{0}$ and ${\beta }_{1}$. This term is added to the variance formula for DTR1 and DTR2 but subtracted for DTR3 and DTR4. The covariances between ${\beta }_{0}$ and ${\beta }_{2}$, and ${\beta }_{1}$ and ${\beta }_{2}$ were approximately zero, thus having negligible impact on the SEs (see Variance Components, Additional file 3 for detailed calculations and Figure S2, Additional file 1).

We observed smaller SEs for MI compared to CCA when estimating the mean outcome for DTR1 (${\theta }_{1}$) and DTR2 (${\theta }_{2}$) across all scenarios (Fig. 4). When estimating the mean outcome for DTR3 (${\theta }_{3}$) and DTR4 (${\theta }_{4}$), the SEs were similar for CCA and MI across all scenarios, except for in missing data scenario 4 (m-DAG 4, Fig. 2), where the SEs for CCA were smaller than for MI.

Across all scenarios, the SEs for both CCA and MI increased with higher proportions of missingness when estimating $\beta$ and $\theta$ parameters. In general, SEs were similar across strong and weak associations between the missing indicators and their predictors of missingness for both CCA and MI. An exception was observed when estimating the mean outcomes for DTR3 (${\theta }_{3}$) and DTR4 (${\theta }_{4}$) under MI in missing data scenario 4 with 40% missingness, where the SEs were higher for stronger associations compared to weaker associations with missing data.

### Coverage

For missing data scenario 1 (m-DAG1, Fig. 2), the coverage for all $\beta$ and $\theta$ parameters were close to the nominal 95% (see Fig. 5 and Figure S3, Additional file 1).

For missing data scenario 4 (m-DAG 4, Fig. 2), where stage 2 treatment was missing dependent on stage 1 intermediate outcome, we observed under-coverage for CCA when estimating the mean outcome for all 4 DTRs ($\theta$) (Fig. 5). We also observed under-coverage for CCA when estimating the mean outcome for DTR1 (${\theta }_{1}$) and DTR3 (${\theta }_{3}$) in missing data scenario 2 (mDAG2, Fig. 2) and when estimating the mean outcome for DTR1 (${\theta }_{1}$) and DTR2 (${\theta }_{2}$) in missing data scenario 3 (mDAG3, Fig. 2). For MI, we observed close to 95% coverage for most scenarios, except when estimating the mean outcome for DTR1 (${\theta }_{1}$) and DTR2 (${\theta }_{2}$), when the intermediate outcome at stage 1 was missing dependent on baseline variables and stage 1 treatment (missing data scenario 3, m-DAG3, Fig. 2).

The problem of under-coverage observed when estimating $\beta$ and $\theta$ parameters (described above) was worse for CCA compared to MI. This was further exacerbated with the increase in the proportion of missingness and the strength of the associations between the missing indicators and their predictors of missingness.

## Discussion

In this simulation study, we evaluated the performance of CCA and MI for handling missing data when IPW was used to estimate the mean outcome of DTRs embedded within a typical two-stage SMART where only non-responders were re-randomised at stage 2. We found that MI showed close to zero bias for all scenarios, except when estimating the mean outcome for DTRs when the stage 1 intermediate outcome was missing dependent on baseline variables and stage 1 treatment. In comparison, CCA generally showed greater bias than MI for all other missing data scenarios when any data missing were dependent on other variables. Both CCA and MI produced comparable empirical standard errors, with MI showing slightly lower values across most scenarios.

Our findings demonstrate that when IPW was used for the estimation of DTR mean outcomes, MI produced unbiased results when handling missing data for most scenarios explored. However, we acknowledge that MI could lead to bias in other missing data scenarios. For example, when the probability of the outcome being missing is dependent on the outcome itself. This is consistent with prior literature where MI has shown an advantage over CCA [17, 19, 31] for standard single-stage randomised trials in particular missing outcome data scenarios [31] and improved precision especially when auxiliary variables (additional variables not included in the analysis model but associated with the missing data) were included in the imputation model [18, 32].

Currently, there is limited research on missing data methods in SMARTs. Shortreed et al. [21] proposed a MI strategy to handle missing data in SMARTs and applied this method to data from the Clinical Antipsychotic Trials of Intervention and Effectiveness (CATIE) study. [33] Using weighted regression to estimate the mean outcome for each DTR, they found that CCA estimates were systematically lower and more variable compared to MI. Based on the challenges Shortreed et al. [21] identified regarding missing data in SMARTs, we previously conducted a simulation study evaluating CCA and MI performance in a simple two-stage SMART where all participants were re-randomised at stage 2, focusing on the estimation of the optimal DTR using Q-learning [22]. In that study, we found that MI performed poorly compared to CCA when stage 2 intervention effect varied between participants, possibly due to incompatibilities between the imputation and analysis models introduced by Q-learning’s backward induction nature. In the present study, we focused on a prototypical two-stage SMART design where randomisation to interventions at stage 2 depends on the individual’s response to stage 1 intervention, as this design is more commonly used in practice. We used IPW to estimate the DTR mean outcomes, allowing researchers to compare the different sequences of treatment decisions. Our simulation results showed that in general MI produced minimal bias, consistent with Shortreed et al.’s findings [21] where CCA estimates were slightly more variable than MI estimates.

Our paper examined four common missing data scenarios that we anticipate might occur in a two-stage two-treatment SMART. We used m-DAGs [16] to outline the causal relationships between variables with missing data and their respective predictors of missingness. As mentioned in our previous study [22], it is important to use m-DAGs to understand and prespecify the potential reasons for missing data in the multiple stages of SMARTs, and additionally, the m-DAGs can guide selecting the appropriate method to handle the missing data [16, 23]. However, our results are limited to the four missing data scenarios considered, and these scenarios may not capture the more complex patterns of missingness that may occur in SMARTs in practice (e.g., when missingness in the outcome is dependent on the outcome itself). Further research is needed to explore more complex missing data scenarios and different SMART designs (e.g., imbalanced design where randomisation at stage 2 depends on both treatment and outcome at stage 1). Although this study has focused on an evaluation of CCA and MI, there are other approaches that could have been used to handle missing data such as IPW or likelihood-based methods [21]. Evaluation of these alternative approaches in the SMART context would provide valuable guidance to researchers on missing data methods for SMARTs. However, we chose to focus on CCA and MI as CCA is the most commonly used method to handle missing data [34], and MI is generally more efficient than IPW [35] and easier to implement than likelihood-based methods [36]. Another direction of research could be to develop practical imputation strategies that are tailored to the SMART design, such as using stage-specific imputation models or performing sequential imputation.

## Conclusion

In conclusion, our simulation study focused on handling missing data in a prototypical SMART where only the non-responders at stage 1 are re-randomised at stage 2. The results demonstrate that MI generally leads to negligible bias in the missing data scenarios explored compared to CCA when estimating the mean outcomes of DTRs using IPW.

## Supplementary Information

Additional file 1.

Additional file 2.

Additional file 3.

## Data Availability

The statistical computing code for this simulation study is available on GitHub https://github.com/jessicaxu3205/SMART_missing_IPW.

## Abbreviations

CATIE: Clinical Antipsychotic Trials of Intervention and Effectiveness
CCA: Complete case analysis
DTRs: Dynamic Treatment Regimens
IPW: Inverse probability weighting
m-DAGs: Missing data directed acyclic graphs
MI: Multiple imputation
MSM: Marginal structural model
OR: Odds ratio
SMARTs: Sequential multiple assignment randomised trials
STEP: Staged treatment in early psychosis

## References

[1] Petersen ML, Deeks SG, van der Laan MJ. Individualized treatment rules: generating candidate clinical trials. Stat Med. 2007;26:4578–601. 10.1002/sim.2888. 17450501 10.1002/sim.2888 PMC2442037

[2] Mahar RK, McGuinness MB, Chakraborty B, et al. A scoping review of studies using observational data to optimise dynamic treatment regimens. BMC Med Res Methodol. 2021;21(1):39. 10.1186/s12874-021-01211-2. 33618655 10.1186/s12874-021-01211-2 PMC7898728

[3] Almirall D, Compton SN, Gunlicks-Stoessel M, et al. Designing a pilot sequential multiple assignment randomized trial for developing an adaptive treatment strategy. Stat Med. 2012;31:1887–902. 10.1002/sim.4512. 22438190 10.1002/sim.4512 PMC3399974

[4] Laber EB, Lizotte DJ, Qian M, et al. Dynamic treatment regimes: technical challenges and applications. Electron J Stat. 2014;8(1):1225–72. 10.1214/14-ejs920. 25356091 10.1214/14-ejs920 PMC4209714

[5] Murphy SA. An experimental design for the development of adaptive treatment strategies. Stat Med. 2005;24:1455–81. 10.1002/sim.2022. 15586395 10.1002/sim.2022

[6] Kidwell KM, Almirall D. Sequential, Multiple Assignment, Randomized Trial Designs. JAMA. 2023;329:336–7. 10.1001/jama.2022.24324. 36692577 10.1001/jama.2022.24324 PMC10061579

[7] Freeman NLB, Browder SE, Rowland BT, et al. Design characteristics of Sequential Multiple Assignment Randomized Trials (SMARTs) for human health: a scoping review of studies between 2009–2024. *medRxiv* 2025 20250608. 10.1101/2025.06.06.25329149. 10.1136/bmjopen-2025-105506 PMC12766822 41469071

[8] Nahum-Shani I, Qian M, Almirall D, et al. Q-learning: a data analysis method for constructing adaptive interventions. Psychol Methods. 2012;17:478–94. 10.1037/a0029373. 23025434 10.1037/a0029373 PMC3747013

[9] Ertefaie A, Wu T, Lynch KG, et al. Identifying a set that contains the best dynamic treatment regimes. Biostatistics. 2016;17(1):135–48. 10.1093/biostatistics/kxv025. 26243172 10.1093/biostatistics/kxv025 PMC4679070

[10] Whiston A, Kidwell KM, O’Reilly S, et al. The use of sequential multiple assignment randomized trials (SMARTs) in physical activity interventions: a systematic review. BMC Med Res Methodol. 2024;24(1):308. 10.1186/s12874-024-02439-4. 39701990 10.1186/s12874-024-02439-4 PMC11658464

[11] Montoya LM, Kosorok MR, Geng EH, et al. Efficient and robust approaches for analysis of sequential multiple assignment randomized trials: illustration using the ADAPT-R trial. Biometrics. 2023;79:2577–91. 10.1111/biom.13808. 36493463 10.1111/biom.13808 PMC10424093

[12] Nahum-Shani I, Qian M, Almirall D, et al. Experimental design and primary data analysis methods for comparing adaptive interventions. Psychol Methods. 2012;17:457–77. 10.1037/a0029372. 23025433 10.1037/a0029372 PMC3825557

[13] Shortreed SM, Laber E, Lizotte DJ, et al. Informing sequential clinical decision-making through reinforcement learning: an empirical study. Mach Learn. 2011;84:109–36. 10.1007/s10994-010-5229-0. 21799585 10.1007/s10994-010-5229-0 PMC3143507

[14] McGorry PD, Mei C, Amminger GP, et al. A sequential adaptive intervention strategy targeting remission and functional recovery in young people at ultrahigh risk of psychosis: the staged treatment in early psychosis (STEP) sequential multiple assignment randomized trial. JAMA Psychiatry. 2023;80:875–85. 10.1001/jamapsychiatry.2023.1947. 37378974 10.1001/jamapsychiatry.2023.1947 PMC10308298

[15] Kasari C, Kaiser A, Goods K, et al. Communication interventions for minimally verbal children with autism: a sequential multiple assignment randomized trial. J Am Acad Child Adolesc Psychiatry. 2014;53:635–46. 10.1016/j.jaac.2014.01.019. 24839882 10.1016/j.jaac.2014.01.019 PMC4030683

[16] Lee KJ, Carlin JB, Simpson JA, et al. Assumptions and analysis planning in studies with missing data in multiple variables: moving beyond the MCAR/MAR/MNAR classification. Int J Epidemiol. 2023;52:1268–75. 10.1093/ije/dyad008. 36779333 10.1093/ije/dyad008 PMC10396404

[17] Sterne JA, White IR, Carlin JB, et al. Multiple imputation for missing data in epidemiological and clinical research: potential and pitfalls. BMJ. 2009;338:b2393. 10.1136/bmj.b2393. 19564179 10.1136/bmj.b2393 PMC2714692

[18] Lee KJ, Simpson JA. Introduction to multiple imputation for dealing with missing data. Respirology. 2014;19(2):162–7. 10.1111/resp.12226. 24372814 10.1111/resp.12226

[19] Sullivan TR, White IR, Salter AB, et al. Should multiple imputation be the method of choice for handling missing data in randomized trials? Stat Methods Med Res. 2018;27:2610–26. 10.1177/0962280216683570. 28034175 10.1177/0962280216683570 PMC5393436

[20] Austin PC, White IR, Lee DS, et al. Missing data in clinical research: a tutorial on multiple imputation. Can J Cardiol. 2021;37:1322–31. 10.1016/j.cjca.2020.11.010. 33276049 10.1016/j.cjca.2020.11.010 PMC8499698

[21] Shortreed SM, Laber E, Scott Stroup T, et al. A multiple imputation strategy for sequential multiple assignment randomized trials. Stat Med. 2014;33:4202–14. 10.1002/sim.6223. 24919867 10.1002/sim.6223 PMC4184954

[22] Xu J, De Silva AP, Lee KJ, et al. Optimising dynamic treatment regimens using sequential multiple assignment randomised trials data with missing data. BMC Med Res Methodol 2025;25:162. 20250701. 10.1186/s12874-025-02595-1. 10.1186/s12874-025-02595-1 PMC12211643 40597702

[23] Moreno-Betancur M, Lee KJ, Leacy FP, et al. Canonical causal diagrams to guide the treatment of missing data in epidemiologic studies. Am J Epidemiol. 2018;187:2705–15. 10.1093/aje/kwy173. 30124749 10.1093/aje/kwy173 PMC6269242

[24] Morris TP, White IR, Crowther MJ. Using simulation studies to evaluate statistical methods. Stat Med. 2019;38(11):2074–102. 10.1002/sim.8086. 30652356 10.1002/sim.8086 PMC6492164

[25] NeCamp T, Kilbourne A, Almirall D. Comparing cluster-level dynamic treatment regimens using sequential, multiple assignment, randomized trials: regression estimation and sample size considerations. Stat Methods Med Res. 2017;26:1572–89. 10.1177/0962280217708654. 28627310 10.1177/0962280217708654 PMC5802435

[26] Tsiatis AA, Davidian M, Holloway ST, et al. Dynamic treatment regimes: statistical methods for precision medicine. 1st ed. New York: Chapman and Hall/CRC; 2019.

[27] Murphy SA. Optimal dynamic treatment regimes. J R Stat Soc Series B Stat Methodol. 2003;65:331–55. 10.1111/1467-9868.00389.

[28] Kidwell KM, Seewald NJ, Tran Q, et al. Design and analysis considerations for comparing dynamic treatment regimens with binary outcomes from sequential multiple assignment randomized trials. J Appl Stat. 2018;45:1628–51. 10.1080/02664763.2017.1386773. 30555200 10.1080/02664763.2017.1386773 PMC6290910

[29] Azur MJ, Stuart EA, Frangakis C, et al. Multiple imputation by chained equations: what is it and how does it work? Int J Methods Psychiatr Res. 2011;20:40–9. 10.1002/mpr.329. 21499542 10.1002/mpr.329 PMC3074241

[30] White IR, Royston P, Wood AM. Multiple imputation using chained equations: issues and guidance for practice. Stat Med. 2011;30:377–99. 10.1002/sim.4067. 21225900 10.1002/sim.4067

[31] Groenwold RH, Donders AR, Roes KC, et al. Dealing with missing outcome data in randomized trials and observational studies. Am J Epidemiol. 2012;175:210–7. 10.1093/aje/kwr302. 22262640 10.1093/aje/kwr302

[32] Hughes RA, Heron J, Sterne JAC, et al. Accounting for missing data in statistical analyses: multiple imputation is not always the answer. Int J Epidemiol. 2019;48:1294–304. 10.1093/ije/dyz032. 30879056 10.1093/ije/dyz032 PMC6693809

[33] Stroup TS, McEvoy JP, Swartz MS, et al. The National Institute of Mental Health Clinical Antipsychotic Trials of Intervention Effectiveness (CATIE) Project: schizophrenia trial design and protocol development. Schizophr Bull. 2003;29:15–31. 10.1093/oxfordjournals.schbul.a006986. 12908658 10.1093/oxfordjournals.schbul.a006986

[34] Bell ML, Fiero M, Horton NJ, et al. Handling missing data in RCTs; a review of the top medical journals. BMC Med Res Methodol. 2014;14(1):118. 10.1186/1471-2288-14-118. 25407057 10.1186/1471-2288-14-118 PMC4247714

[35] Seaman SR, White IR. Review of inverse probability weighting for dealing with missing data. Stat Methods Med Res. 2013;22:278–95. 10.1177/0962280210395740. 21220355 10.1177/0962280210395740

[36] Collins LM, Schafer JL, Kam CM. A comparison of inclusive and restrictive strategies in modern missing data procedures. Psychol Methods. 2001;6:330–51. 11778676

