# Supplementary material for: Inverse probability weighted estimation of dynamic treatment regimen means in sequential multiple assignment randomised trials with missing data: a simulation study
Source: Trials. 2026 Jan 30;27:178. doi: 10.1186/s13063-026-09493-x (PMC12930820; doi:10.1186/s13063-026-09493-x)
Supplement: Supplementary file 1 — Additional file 1. [file 13063_2026_9493_MOESM1_ESM.docx]

**Additional file 1**

Figures S1- S3 summarise the performance of complete case analysis (CCA) and multiple imputation (MI) for estimating the parameters of the marginal structural models (MSM) ($\beta s$) across the different missing data scenarios described in Figure 2 of the main text.

***Bias***

In the estimation of $\beta_{0}$, CCA showed greater bias than MI (for which the bias was minimal or close to zero) for all missing data scenarios except for missing data scenario 1, where the outcome was missing not dependent on any variables (Figure S1). Both MI and CCA showed minimal bias in the estimation of $\beta_{1}$ for all missing data scenarios except for missing data scenario 3, where the intermediate outcome at stage 1 was missing dependent on baseline variables and stage 1 treatment (m-DAG3, Main text: Figure 2). For all missing data scenarios both MI and CCA showed minimal bias for the estimation of $\beta_{2}$ except for missing data scenario 2 (outcome was missing dependent on the intermediate outcome at stage 1 and the treatment given in stage 2) where CCA showed greater bias than MI.

***Empirical and Model-based Standard Errors***

For all scenarios, empirical SEs for $\beta$ parameters were similar to their corresponding model-based SEs (Figure S2 and Table S1, Additional file 2). In general, the SEs for the $\beta$ parameters were similar for CCA and MI when there was 20% missingness. With 40% missingness, we observed smaller SEs for MI compared to CCA for the $\beta$ parameters across all scenarios, except for in missing data scenario 4 (m-DAG4, Main text: Figure 2), where the SEs for $\beta_{2}$ were smaller for CCA compared to MI (Figure S2, Additional file 1).

***Coverage***

For missing data scenario 1 (m-DAG1, Main text: Figure 2), the coverage for all $\beta$ parameters were around the nominal 95% (Figure S3, Additional file 1). We observed low coverage in the estimation of $\beta_{0}$ for CCA in all other missing data scenarios (m-DAG2-4, Main text: Figure 2) and for MI in missing data scenario 3 (m-DAG3, Main text: Figure 2). We also observed low coverage for $\beta_{1}$ for CCA and MI in missing data scenarios 3 and 4 (m-DAG3-4, Main text: Figure 2). The coverage for $\beta_{2}$ was around 95% for both CCA and MI in all missing data scenarios, except for when the stage 2 outcome was missing dependent on the stage 1 intermediate outcome and stage 2 treatment (missing data scenario 2, m-DAG2, Main text: Figure 2) there was low coverage for CCA.


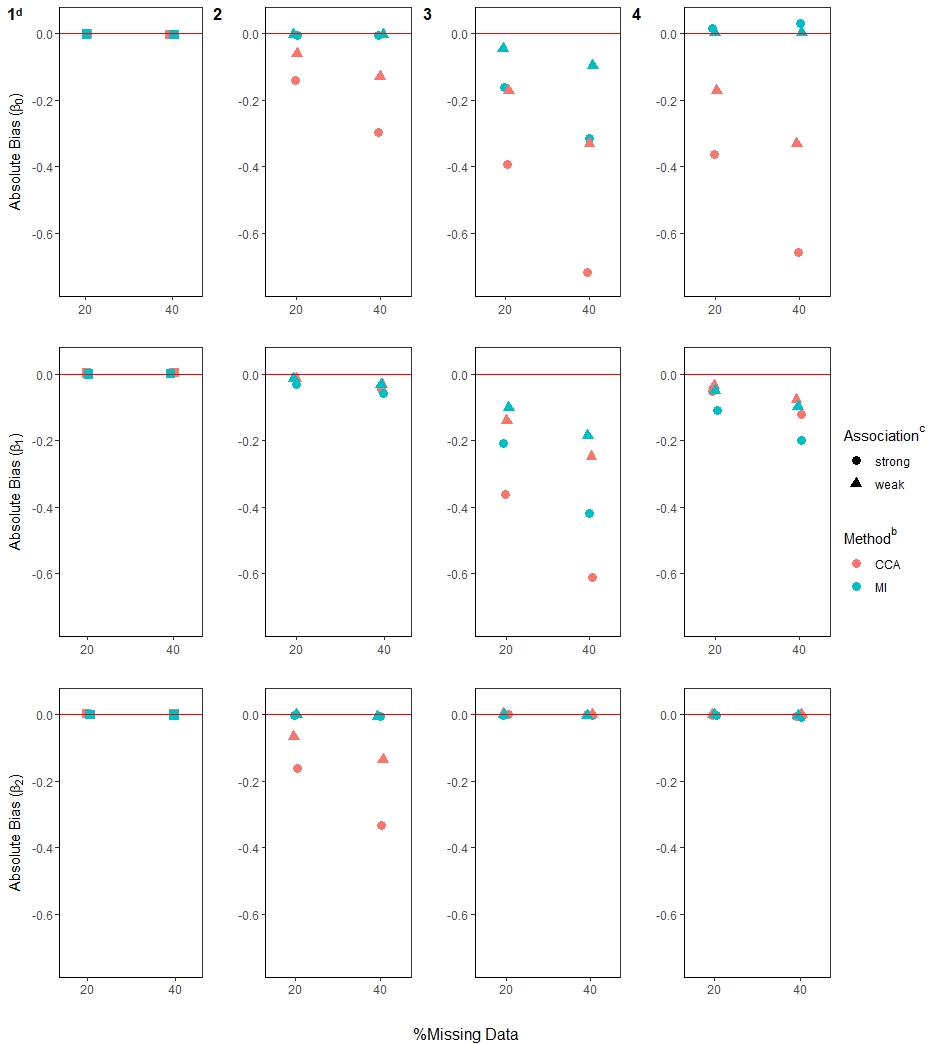


Figure S1: Bias in estimating the $\beta$ parameters^a^, presented by rows, in the 4 missing data scenarios^b^ (depicted in the m-DAGs in Figure 2).

Footnotes: ^a^ True values are $\beta^{*}=\left( 1.25, -0.15, 0.03 \right)$.

^b^ Complete case analysis (CCA) and multiple imputation (MI) were used to handle missing data, where 20% or 40% had incomplete data under the four missing data scenarios (presented by columns) described in the Missingness in SMART designs section, see Figure 2.

^c^ For a weak association between the missing indicator and other variables (as described below) an OR of 1.6 was used; and for a strong association an OR of 3 was used. The other variables used in missing data scenario: 2)$O_{2}\to M_{Y}$ and $A_{2}\to M_{Y}$; 3) $A_{1}\to M_{O2}$ and $O_{1}\to M_{O2}$; and 4)$O_{2}\to M_{A2}$.

^d^ For m-DAG 1, where only stage 2 outcome was missing and the missingness was not dependent on any variables, a square symbol is used.

^e^ Monte Carlo errors ranged from 0.0026–0.0053.


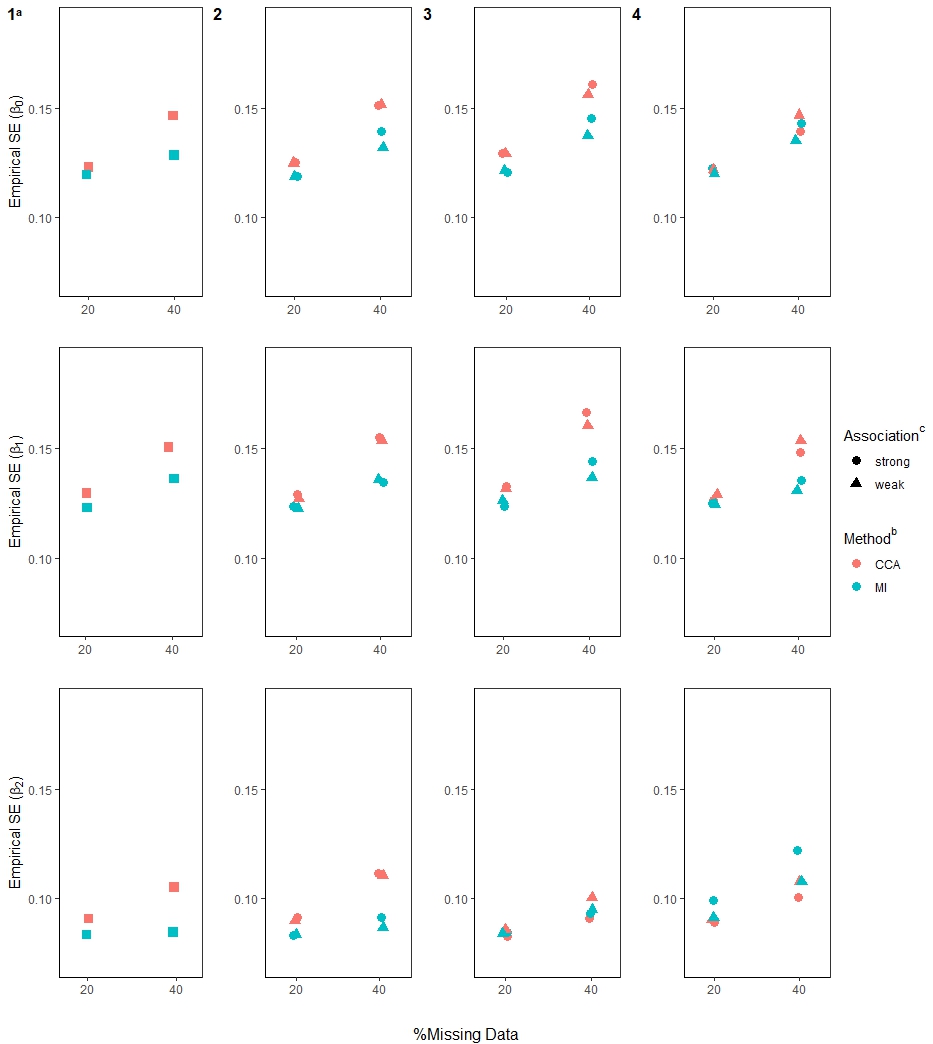


Figure S2: Empirical standard errors (SEs) for the $\beta$ parameters, presented by rows, in the 4 missing data scenarios^b^ (depicted in the m-DAGs in Figure 2).

Footnotes: ^a^ For m-DAG 1, where only stage 2 outcome was missing and the missingness was not dependent on any variables, a square symbol is used.

^b^ Complete case analysis (CCA) and multiple imputation (MI) were used to handle missing data, where 20% or 40% had incomplete data under the four missing data scenarios (presented by columns) described in the Missingness in SMART designs section, see Figure 2.

^c^ For a weak association between the missing indicator and other variables (as described below) an OR of 1.6 was used; and for a strong association an OR of 3 was used. The other variables used in missing data scenario: 2)$O_{2}\to M_{Y}$ and $A_{2}\to M_{Y}$; 3) $A_{1}\to M_{O2}$ and $O_{1}\to M_{O2}$; and 4)$O_{2}\to M_{A2}$.

^d^ Monte Carlo errors ranged from 0.0018–0.0037.


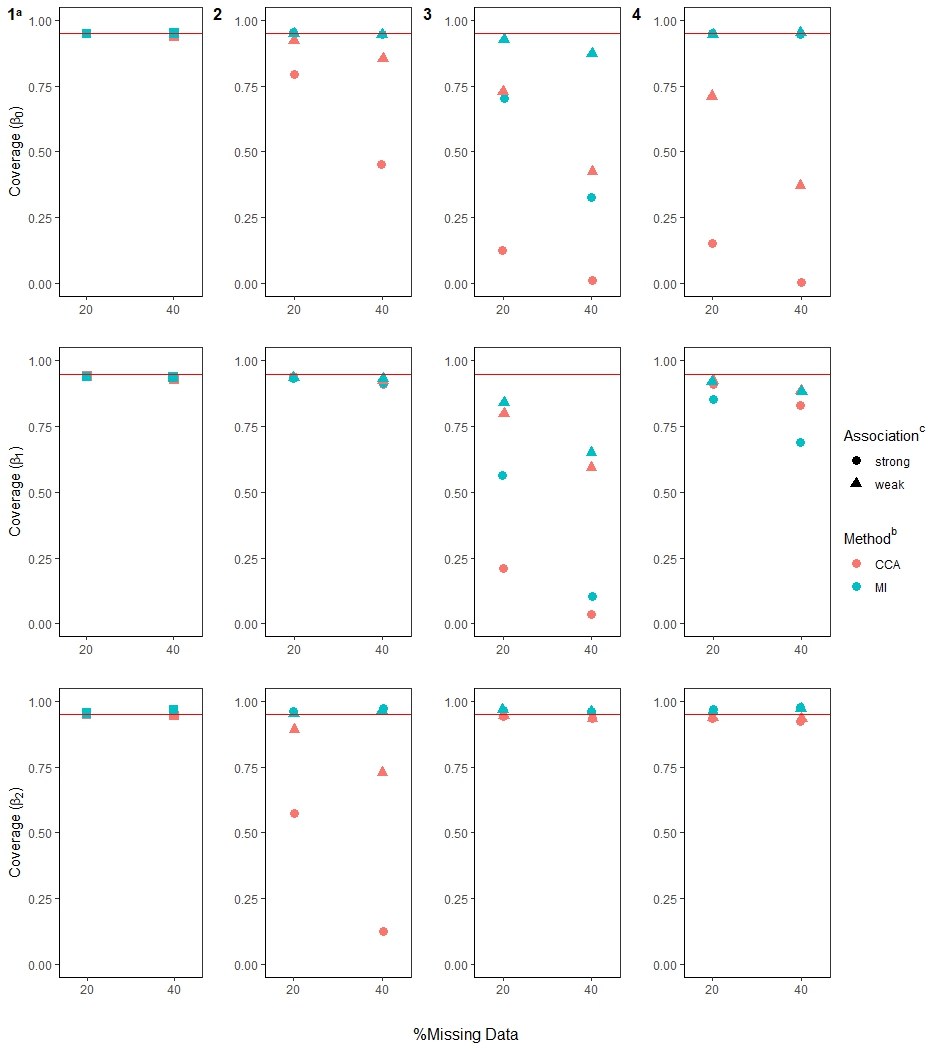


Figure S3: Coverage for the $\beta$ parameters, presented by rows, in the 4 missing data scenarios^b^ (depicted in the m-DAGs in Figure 2).

Footnotes: ^a^ For m-DAG 1, where only stage 2 outcome was missing and the missingness was not dependent on any variables, a square symbol is used.

^b^ Complete case analysis (CCA) and multiple imputation (MI) were used to handle missing data, where 20% or 40% had incomplete data under the four missing data scenarios (presented by columns) described in the Missingness in SMART designs section, see Figure 2.

^c^ For a weak association between the missing indicator and other variables (as described below) an OR of 1.6 was used; and for a strong association an OR of 3 was used. The other variables used in missing data scenario: 2)$O_{2}\to M_{Y}$ and $A_{2}\to M_{Y}$; 3) $A_{1}\to M_{O2}$ and $O_{1}\to M_{O2}$; and 4)$O_{2}\to M_{A2}$.

^d^ Monte Carlo errors ranged from 0.0020–0.0157.
