# Supplementary material for: Inverse probability weighted estimation of dynamic treatment regimen means in sequential multiple assignment randomised trials with missing data: a simulation study
Source: Trials. 2026 Jan 30;27:178. doi: 10.1186/s13063-026-09493-x (PMC12930820; doi:10.1186/s13063-026-09493-x)
Supplement: Supplementary file 2 — Additional file 2. [file 13063_2026_9493_MOESM2_ESM.docx]

**Additional file 2**

Table S1: Model-based standard errors (SEs) for the parameters of the marginal structural model (MSM) ($\beta$) and the dynamic treatment regimens (DTR) mean outcomes ($\theta$).

| **Methods** | **% Missingness** | **Association** |  | |  | **Model-based SEs** | |  |  | |  |
| --- | --- | --- | --- | --- | --- | --- | --- | --- | --- | --- | --- |
|  |  |  | $\boldsymbol{\beta}$ **parameters** | | | | $\boldsymbol{\theta}$ **parameters** | | | | |
|  |  |  | $\boldsymbol{\beta}_{\boldsymbol{0}}$ | $\boldsymbol{\beta}_{\boldsymbol{1}}$ | | $\boldsymbol{\beta}_{\boldsymbol{2}}$ | $\boldsymbol{\theta}_{\boldsymbol{1}}$ | $\boldsymbol{\theta}_{\boldsymbol{2}}$ | $\boldsymbol{\theta}_{\boldsymbol{3}}$ | $\boldsymbol{\theta}_{\boldsymbol{4}}$ | |
| **Missing data scenario 1 (m-DAG1)** | | | | | | | | | | | |
| CCA | 20 | NA | 0.129 | | 0.129 | 0.092 | 0.246 | 0.245 | 0.152 | | 0.150 |
| MI | 20 | NA | 0.121 | | 0.120 | 0.087 | 0.221 | 0.219 | 0.158 | | 0.157 |
| CCA | 40 | NA | 0.149 | | 0.149 | 0.106 | 0.285 | 0.283 | 0.175 | | 0.173 |
| MI | 40 | NA | 0.130 | | 0.130 | 0.093 | 0.227 | 0.226 | 0.183 | | 0.182 |
| **Missing data scenario 2 (m-DAG2)** | | | | | | | | | | | |
| CCA | 20 | strong | 0.130 | | 0.128 | 0.093 | 0.251 | 0.240 | 0.166 | | 0.142 |
| MI | 20 | strong | 0.123 | | 0.121 | 0.091 | 0.224 | 0.220 | 0.176 | | 0.149 |
| CCA | 40 | strong | 0.150 | | 0.147 | 0.107 | 0.289 | 0.272 | 0.204 | | 0.156 |
| MI | 40 | strong | 0.139 | | 0.131 | 0.103 | 0.243 | 0.225 | 0.220 | | 0.171 |
| CCA | 20 | weak | 0.129 | | 0.128 | 0.092 | 0.248 | 0.242 | 0.158 | | 0.146 |
| MI | 20 | weak | 0.121 | | 0.121 | 0.089 | 0.222 | 0.220 | 0.165 | | 0.153 |
| CCA | 40 | weak | 0.150 | | 0.149 | 0.107 | 0.289 | 0.278 | 0.190 | | 0.166 |
| MI | 40 | weak | 0.132 | | 0.131 | 0.096 | 0.232 | 0.225 | 0.197 | | 0.177 |
| **Missing data scenario 3 (m-DAG3)** | | | | | | | | | | | |
| CCA | 20 | strong | 0.129 | | 0.129 | 0.082 | 0.248 | 0.246 | 0.138 | | 0.137 |
| MI | 20 | strong | 0.115 | | 0.114 | 0.092 | 0.219 | 0.218 | 0.148 | | 0.147 |
| CCA | 40 | strong | 0.159 | | 0.159 | 0.088 | 0.310 | 0.307 | 0.148 | | 0.147 |
| MI | 40 | strong | 0.129 | | 0.124 | 0.102 | 0.241 | 0.239 | 0.165 | | 0.164 |
| CCA | 20 | weak | 0.130 | | 0.130 | 0.088 | 0.251 | 0.250 | 0.145 | | 0.143 |
| MI | 20 | weak | 0.119 | | 0.119 | 0.093 | 0.224 | 0.222 | 0.154 | | 0.153 |
| CCA | 40 | weak | 0.155 | | 0.155 | 0.098 | 0.300 | 0.298 | 0.161 | | 0.159 |
| MI | 40 | weak | 0.128 | | 0.127 | 0.105 | 0.237 | 0.236 | 0.177 | | 0.176 |
| **Missing data scenario 4 (m-DAG4)** | | | | | | | | | | | |
| CCA | 20 | strong | 0.124 | | 0.125 | 0.087 | 0.236 | 0.234 | 0.149 | | 0.147 |
| MI | 20 | strong | 0.126 | | 0.124 | 0.114 | 0.232 | 0.230 | 0.186 | | 0.186 |
| CCA | 40 | strong | 0.144 | | 0.143 | 0.097 | 0.268 | 0.266 | 0.174 | | 0.171 |
| MI | 40 | strong | 0.144 | | 0.135 | 0.142 | 0.253 | 0.252 | 0.234 | | 0.233 |
| CCA | 20 | weak | 0.128 | | 0.128 | 0.091 | 0.244 | 0.242 | 0.152 | | 0.151 |
| MI | 20 | weak | 0.122 | | 0.122 | 0.101 | 0.227 | 0.225 | 0.171 | | 0.170 |
| CCA | 40 | weak | 0.148 | | 0.148 | 0.104 | 0.279 | 0.278 | 0.178 | | 0.176 |
| MI | 40 | weak | 0.134 | | 0.132 | 0.121 | 0.240 | 0.239 | 0.208 | | 0.207 |

Footnotes: ^a^ Complete case analysis (CCA) and multiple imputation (MI) were used to handle missing data, where 20% or 40% had incomplete data under the four missing data scenarios described in the Missingness in SMART designs section, see Figure 2 in main text.

^b^ For a weak association between the missing indicator and other variables (as described below) an OR of 1.6 was used; and for a strong association an OR of 3 was used. The other variables used in missing data scenario: 2)$O_{2}\to M_{Y}$ and $A_{2}\to M_{Y}$; 3) $A_{1}\to M_{O2}$ and $O_{1}\to M_{O2}$; and 4)$O_{2}\to M_{A2}$.

^c^ The DTR mean outcomes ($\theta_{k}$) were derived from: $\theta_{k}=\beta_{0}+\beta_{1}A_{1}+\beta_{2}A_{2}^{NR}$, where $k=1,\ldots, 4,$for the four combinations of $(A_{1},A_{2}^{NR})$: $\theta_{1}\to\left( 1,1 \right); \theta_{2}\to\left( 1,-1 \right);\theta_{3}\to\left( -1,1 \right);$ and $\theta_{4}\to\left( -1,-1 \right)$.
