# Supplementary material for: Inverse probability weighted estimation of dynamic treatment regimen means in sequential multiple assignment randomised trials with missing data: a simulation study
Source: Trials. 2026 Jan 30;27:178. doi: 10.1186/s13063-026-09493-x (PMC12930820; doi:10.1186/s13063-026-09493-x)
Supplement: Supplementary file 3 — Additional file 3. [file 13063_2026_9493_MOESM3_ESM.docx]

**Additional file 3**

The mean outcome $\theta_{k}$ for each dynamic treatment regimen (DTR) ($k$) were estimated by the following model where A_1_ and A_2_ represent the treatments at stages 1 and 2 respectively, and superscript NR represents non-responders:

$$\theta_{k}=\beta_{0}+\beta_{1}A_{1}+\beta_{2}A_{2}^{NR}$$

Therefore, the variance of $\theta$ was dependent on the variance and covariances of the $\beta$s:

$$Var\left( \theta\right)=Var\left( \beta_{0} \right)+A_{1}^{2}Var\left( \beta_{1} \right)+{A_{2}^{NR}}^{2}Var\left( \beta_{2} \right)+2A_{1}Cov\left( \beta_{0},\beta_{1} \right)+2{A_{2}^{NR}}^{2}Cov\left( \beta_{0},\beta_{2} \right)+2A_{1}A_{2}^{NR}Cov(\beta_{1},\beta_{2})$$

From the simulations of 1000 datasets the empirical variances and covariances were:

$$Var\left( \beta_{0} \right)={0.11}^{2}, Var\left( \beta_{1} \right)={0.12}^{2}, Var\left( \beta_{2} \right)={0.08}^{2},$$

$$Cov\left( \beta_{0},\beta_{1} \right)=0.00748, Cov\left( \beta_{0},\beta_{2} \right)=0.00012, Cov\left( \beta_{1},\beta_{2} \right)=0.00008$$

Table S2: Variance of the four dynamic treatment regimen (DTR) mean outcomes ($\theta$) for each combination of treatment $A_{1}$ and $A_{2}^{NR}$.

| DTR ($\boldsymbol{A}_{\boldsymbol{1}}$, $\boldsymbol{A}_{\boldsymbol{2}}^{\boldsymbol{NR}}$) | Variance Formula |
| --- | --- |
| DTR1  ($\boldsymbol{A}_{\boldsymbol{1}}\boldsymbol{=1}$, $\boldsymbol{A}_{\boldsymbol{2}}^{\boldsymbol{NR}}\boldsymbol{=1}$) | $Var\left( \theta_{1} \right)=\left[ Var\left( \beta_{0} \right)+Var\left( \beta_{1} \right)+Var\left( \beta_{2} \right) \right]\boldsymbol{+2}\boldsymbol{Cov}\left( \boldsymbol{\beta}_{\boldsymbol{0}}\boldsymbol{,}\boldsymbol{\beta}_{\boldsymbol{1}} \right)+2Cov\left( \beta_{0},\beta_{2} \right)+2Cov\left( \beta_{1},\beta_{2} \right)=0.048 ({\text{or} 0.22}^{2})$ |
| DTR2  ($\boldsymbol{A}_{\boldsymbol{1}}\boldsymbol{=1}$, $\boldsymbol{A}_{\boldsymbol{2}}^{\boldsymbol{NR}}\boldsymbol{=-1}$) | $Var\left( \theta_{2} \right)=[Var\left( \beta_{0} \right)+Var\left( \beta_{1} \right)+Var\left( \beta_{2} \right)]\boldsymbol{+2}\boldsymbol{Cov}\left( \boldsymbol{\beta}_{\boldsymbol{0}}\boldsymbol{,}\boldsymbol{\beta}_{\boldsymbol{1}} \right)-2Cov\left( \beta_{0},\beta_{2} \right)-2Cov\left( \beta_{1},\beta_{2} \right)=0.047 ({\text{or} 0.22}^{2})$ |
| DTR3  $\boldsymbol{(}\boldsymbol{A}_{\boldsymbol{1}}\boldsymbol{=-1}$, $\boldsymbol{A}_{\boldsymbol{2}}^{\boldsymbol{NR}}\boldsymbol{=1}$) | $Var\left( \theta_{3} \right)=[Var\left( \beta_{0} \right)+Var\left( \beta_{1} \right)+Var\left( \beta_{2} \right)]\boldsymbol{-2}\boldsymbol{Cov}\left( \boldsymbol{\beta}_{\boldsymbol{0}}\boldsymbol{,}\boldsymbol{\beta}_{\boldsymbol{1}} \right)+2Cov\left( \beta_{0},\beta_{2} \right)-2Cov\left( \beta_{1},\beta_{2} \right)=0.018 ({\text{or} 0.13}^{2})$ |
| DTR4  ($\boldsymbol{A}_{\boldsymbol{1}}\boldsymbol{=-1}$, $\boldsymbol{A}_{\boldsymbol{2}}^{\boldsymbol{NR}}\boldsymbol{=-1}$) | $Var\left( \theta_{4} \right)=[Var\left( \beta_{0} \right)+Var\left( \beta_{1} \right)+Var\left( \beta_{2} \right)]\boldsymbol{-2}\boldsymbol{Cov}\left( \boldsymbol{\beta}_{\boldsymbol{0}}\boldsymbol{,}\boldsymbol{\beta}_{\boldsymbol{1}} \right)-2Cov\left( \beta_{0},\beta_{2} \right)+2Cov\left( \beta_{1},\beta_{2} \right)=0.018 ({\text{or} 0.13}^{2})$ |

Footnotes: Variances for DTR1 and DTR2 were similar and variances for DTR3 and DTR4 were similar.
